# Metagenomic analysis of microbial community and function involved in cd-contaminated soil

**DOI:** 10.1186/s12866-018-1152-5

**Published:** 2018-02-13

**Authors:** Gang Feng, Tian Xie, Xin Wang, Jiuyuan Bai, Lin Tang, Hai Zhao, Wei Wei, Maolin Wang, Yun Zhao

**Affiliations:** 1 Key Laboratory of Bio-Resource and Eco-Environment of Ministry of Education, College of life sciences, Sichuan University, No. 24 South Section 1, Yihuan Road, Chengdu, 610065 China; 2 Tibet Academy of Agriculture and Animal Husbandry Sciences, Lhasa, 850002 China; 3 Chengdu Institute of Biology, Chinese Academy of Sciences, Chengdu, 610041 China

**Keywords:** Metagenomic, Microbial community, Cadmium-contaminated, Functional potentials

## Abstract

**Background:**

Soil contaminated with the heavy metal Cadmium (Cd) is a widespread problem in many parts of the world. Based on metagenomic analysis, we investigated the functional potential and structural diversity of the microbial community in Cd-contaminated and non-contaminated soil samples and we explored the associated metabolic pathway network in cluster of orthologous groups (COG) and Kyoto Encyclopedia of Genes and Genomes (KEGG).

**Results:**

The results showed that microorganisms in these soils were quite abundant, and many of them possessed numerous physiological functions. However, Cd-contamination has the potential to reduce the microbial diversity and further alter the community structure in the soil. Notably, function analysis of the crucial microorganisms (e. g. *Proteobacteria, Sulfuricella* and *Thiobacillus*) indicated that these bacteria and their corresponding physiological functions were important for the community to cope with Cd pollution. The COG annotation demonstrated that the predominant category was the microbial metabolism cluster in both soil samples, while the relative abundance of metabolic genes was increased in the Cd-contaminated soil. The KEGG annotation results exhibited that the non-contaminated soil had more genes, pathways, modules, orthologies and enzymes involved in metabolic pathways of microbial communities than the Cd-contaminated soil. The relative abundance of some dominant KEGG pathways increased in the Cd contaminated soil, and they were mostly enriched to the metabolism, biosynthesis and degradation of amino acids, fatty acids and nucleotides, which was related to Cd tolerance of the microorganisms.

**Conclusions:**

Cd-contamination can decrease the taxonomic species of microbes in soil and change the soil microbial composition. The functional pathways involved in the soil change with microbial structure variation, many of which are related to the heavy metal tolerance of soil microbes. The Cd-contaminated soil microbes is a potential resource for exploring cadmium resistant or tolerant bacteria.

**Electronic supplementary material:**

The online version of this article (10.1186/s12866-018-1152-5) contains supplementary material, which is available to authorized users.

## Background

Heavy metal contamination of soil derived from agricultural or industrial activities is one of the most severe environmental pollution issues around the world [1, 2]. During the past several decades, soil contaminated by heavy metals has become a serious phenomenon in China [3], which is not easy to solve because heavy metals are non-degradable and get accumulated indefinitely in the environment [4]. In particular, the mining activities generate high concentrations of heavy metals (Ni, Cd, Pb, Mn, Cu, and Zn) that are exposed in open areas or agricultural soil. For example, high concentration of cadmium (1.8–7.6 mg/kg) has been found in paddy near the mining sites [5]. Moreover, it has been calculated that about 20% of China’s arable lands are being contaminated by heavy metals, and this situation will deteriorated steadily in the near decades due to human activities, especially with respect to the agricultural soils [6]. Heavy metals gathering in soil would cause a series problems, including reducing the microbial biodiversity, soil quality and crops yield, and then threats animals and humans health via the food chain [7, 8].

It is clear that cadmium is not an essential nutrient element but a harmful factor to plant growth and development. Due to its good solubility in soil, cadmium can be absorbed by the crops planted in the soil and affect the crops’ s tomatal opening, transpiration, and photosynthesis [9]. In addition, the oxidative stress is involved in Cd toxicity have been proved in many researches, then increased the oxygen free radical production, or induced the enzymatic and nonenzymatic antioxidants [10–12], which could affect the normal development and the quality of the crops. Cd contamination has also been found to significantly decrease the microbial numbers and diversity in the soil [13, 14], leading to the reduction of soil microbial biomass, the respiration rate of microbes and enzyme activity [15–19], and decreased utilization of carbon [20].

Studies involved in soil microbial community composition could not only help us to explore the potential risks associated with contaminated soils but also provide insight into possible soil remediation strategies using indigenous resistant bacteria. It is well known that the some rhizosphere microorganisms can be used for phytoremediation [21, 22]. Furthermore, as the most active element of the soil ecosystem, microbes can rapidly respond to anthropogenic pressures, making it possible to be an indicator of soil quality and health [23, 24]. However, excess cadmium in soil has been shown to change the taxonomic diversity and function of the native microbial species [25–27]. For example, Hong and Si showed that varying concentrations of heavy metals surrounding an iron mining areas increased bacterial *alpha* diversity and resulted in a shift of the dominant genera [28]. Tonin et al. determined that *Arbuscular Mycorrhizal* (AM) fungi and plants associated with heavy metal tolerance can effectively enhance the heavy metal accumulation of plant roots [29].

To remedy the soil problem, the mechanism of metal resistance should be dissected as clear as possible. However, current knowledge about this is mostly based on cultured microorganisms, which results in less metal resistance determinants to be discovered. Because traditional microbiological techniques only isolate and culture a small proportion (0.1 to 1%) of the microorganisms in soil samples [30–33], therefore obtaining incomplete information about soil bacteria. However, some uncultivable bacteria may confer metal resistance, which was a vital environmental importance because of their potential application in bio-remediation area. Recently, efforts have been tried to identify genes from environmental samples via culture-independent methods, but they were amplified or detected because of their similarity to the previously known genes [34–36], which is invalidate for exploiting novel mechanisms of metal resistance. As the advancement of cultivation-independent metagenomic approaches, it has been used to analysis the soil microbial community [37, 38], and improved our knowledge of the soil microbes and its potential significance. Moreover, the local soil microbial communities are mainly comprised of some dominant species and many other rare taxa [39]. Some of them with a low-abundance may be the novel microbial lineages [40, 41], and could play a crucial role in biogeochemical interactions of the soil-plant system [42–44]. Thus, the wealth of information obtained from the deep metagenomic sequencing makes it possible to capture the genomic information of low-abundance populations and to reveal the multiple activities in soil. It has been successfully introduced into investigating many diverse microbial niches in marine water, grassland soil, human gut, and artificial systems. Additionally, recent attempts have been made to analyze the abundance of bacteria and the associated genes involved in copper resistance from agricultural soils via metagenomic sequencing [45]. It is a useful tool to help us understand the unique characteristics of bacteria and genes associated with heavy metal contaminated soil and to aid in developing novel biological methods that could remedy soil ecosystem damage result from heavy metals.

The environmental troubles in mining areas are mainly related to physical disturbances surrounding spilled mine tailings, emitted dust and metal-containing waste, which results in toxic heavy metals enriched in soils [46]. Thus, microbial communities in these soils are optimal for the discovery of new determinants of metal resistance. Therefore, samples in this study were collected near phosphate rock chemical plants, Sichuan province, China.

In this study, we used metagenomic to analysis the microbial community structure and function in Cd-contaminated soil and reveal the variations of the microbial community, which provided an ecological methodology for investigating potential heavy metal tolerant or resistant bacteria that are able to cope with Cd-polluted soil.

## Methods

### Sample collection

The sample plot was near phosphate rock chemical plants in Shuangsheng town,Shifang County, Sichuan province, China. Two sites, designated site 1 (S_1_) and site 2 (S_2_), were sampled in triplicate from idle soils and without plants, on 7th May, 2015. Our samples were obtained from private land and our sampling was permitted by the land owner. S_1_ consisted of unpolluted soil and S_2_ consisted of Cd-contaminated soil collected next to the sewage outlet of three phosphate rock chemical plants. Samples were collected by removing the topsoil (0 to 5 cm), gathering approximately 500 g of soil at a depth of 5 to 10 cm and then immediately transferring the samples into sterile plastic bags that were kept on ice. Upon returning to the laboratory, half of the soils were immediately frozen in liquid nitrogen and stored at − 80 °C for subsequent molecular analysis. The remaining half was air-dried at room temperature for one week and subsequently sieved through a 100-mesh sifter to remove stones and visible plant fragments. The soils were then stored at 4 °C for further chemical analysis.

### Soil physicochemical analysis

In order to assess the soil quality and the microbial community structure, we closely followed the methods reported by Bloem et al. [47] that suggests indicators for soil quality assessment and microbial analysis.

Physicochemical property of the soil samples was detected at the beginning of the experiment in order to have a baseline for soil characterization. The following metrics were evaluated: soil organic matter (SOM), pH, total phosphorous (TP), total nitrogen (TN), total potassium (TK) and Cd. SOM and TN concentrations were determined using the method described by Sun et al. [48]. We measured the soil pH using an INESA pH meter (Shanghai REX Instrument Factory, Shanghai, China) in a 1:5 suspension of ultrapure water. The TP content was measured using the ammonium molybdate spectrometry [49]. TK content was evaluated via the method in *Soil agricultural chemistry analysis* [50].

All Cd concentrations were determined using inductively coupled plasma mass spectrometry (ICP-MS), digestion of the soil samples with 5 mL HCL at 150 °C for 60 min, 5 mL HNO_3_ at 150 °C for 60 min, 3 mL HF at 150 °C for 10 min, and 3 ml HClO_4_ at 190 °C for 100 min in a graphite digestion apparatus. All mixed soil samples were tested three times; and results are reported as the mean of the data.

### DNA extraction, library construction, and metagenomic sequencing

DNA for metagenomic analysis was extracted from the soil samples using the Power Soil DNA kit (MoBiol Laboratories, Solana Beach, CA), according to the manufacturer’s protocols. The concentration and quality (A_260_/A_280_) of extracted DNA were determined using a NanoDrop ND-2000 spectrophotometer (NanoDrop, Wilmington, DE, USA), and evaluated with a 2% agarose gel. To minimize DNA extraction bias, three replicate DNA isolation of each sample were pooled. Afterwards, DNA was sheared into fragments of approximately 300 bp using an M220 Focused-ultrasonicator (Covaris Inc., Woburn, MA, USA) to build a paired-end library. DNA templates were then pretreated using the TruSeqTM Kit according to the manufacturer’s instructions (http://www.illumina.com/). Following libraries were pooled and loaded onto an Illumina cBot [51]. Pair-end sequencing (2 * 150 bp) was completed at Majorbio Bio-Pharm Technology Co., Ltd. (Shanghai, China) according to the standard protocol (http://www.illumina.com/).

### Selection and assembly of sequencing reads

The 3′-adaptors and 5′-adaptors was stripped. The raw reads were trimmed with a minimum quality score 20 and a minimum length 50 bp to maintain the reliability of the data [52]. Clean reads were then assembled using SOAPdenovo software [52] was used to assembled the clean reads at a Kmer range of 39 to 47. Statistical tests were conducted based on the scaffolds with a length over 500 bp [52]. Scaffolds with a length over 500 bp were then extracted and broken into contigs without gaps [52]. Contigs were used for the following predictions and annotations. The statistical information for the contigs was exhibited in Table 2. To confirm the assembled metagenomic data, all the sequencing reads were mapped to contigs using the Burrows-Wheeler Aligner (http://bio-bwa.sourcefo rge.net/) BWA tool [53]. Pread_contig were calculated as the number of reads successfully aligned to the assembled contigs [52], divided by the total number of clean reads.

### Gene prediction, taxonomy and functional assignment

The open reading frames (ORFs) of contigs and annotations in each sample were predicted with MetaGene Annotator [53]. The predicted ORFs with lengths over 100 bp were retained and translated into amino acid sequences via NCBI ORF finder [53], which were subsequently annotated by BLASTP (BLAST Version 2.2.28+) against NCBI-nr database including SwissProt. The optimized E-value threshold for BLAST Alignment was 1e-5.

In this study, the taxonomy of the metagenomes was assigned by the small-subunit (SSU) rRNA gene tags and predicted proteins [54]. We obtained the SSU rRNA gene tags by align the raw reads with the Silva SSU rRNA database (https://www.arb-silva.de/) with an optimized E-value threshold of 1e-5, alignment length of 100 bp, and identification cutoff 80%. The taxonomic annotation of the samples were obtained by blast against NCBI-nt database. For each protein-coding read, alignment with protein sequences in the NCBI-nr database was performed using the BLAST tool, and results with the best hit to the NCBI microbial taxonomy database were obtained .

The ORFs were aligned using the eggNOG database (evolutionary genealogy of genes: Non-supervised Orthologous Groups, Version 4.0) via BLASTP (BLAST Version 2.2.28+) at the optimized E-value threshold of 1e-5 [54], and the corresponding cluster of orthologous groups of protein (COG) were obtained. A pair-wise statistical comparison of COG classification was carried out using STAMT [54], and the relevance between samples was evaluated using the Mann-Whitney rank sum test in SigmaPlot (Version 12.5, Systat Software, USA). The KEGG pathway annotation was conducted using BLAST search (BLAST Version 2.2.28+) against the Kyoto Encyclopedia of Genes and Genomes database (Kyoto Encyclopedia of Genes and Genomes, KEGG GENES,) at the optimized E-value threshold of 1e-5. The functional annotation of genes was conducted via KOBAS 2.0 (KEGG Orthology Based Annotation System) [53]. Hierarchical clustering analysis of the KEGG annotation results among different samples was carried out using Qiime (version 1.17) and R Programming Language [54].

## Results

### Soil physicochemical background

The initial soil characteristics and Cd concentrations were determined for each site (Table 1). The results showed that the pH value, organic matter, total nitrogen, total phosphorus and total potassium content in the two samples coincide with the local large area soil. The concentrations of Cd in the non-contaminated soil (S_1_) and the contaminated soil (S_2_) were 0.072 and 1.639 mg/kg, respectively. There were no significant differences in all soil characteristics between the two samples except for the Cd concentration (Table 1).

**Table 1:** Physical and chemical properties of soil

Sample	pH	Organic Matter (g/kg)	Total nitrogen (g/kg)	Total phosphorus (g/kg)	Total potassium (g/kg)	Cd concentration (mg/kg)
S_1_	6.66 ± 0.039a	21.07 ± 1.949a	2.13 ± 0.254a	3.62 ± 0.172a	17.83 ± 0.331a	0.072 ± 0.003a
S_2_	6.72 ± 0.024a	18.94 ± 2.589a	1.82 ± 0.176a	3.94 ± 0.030a	17.83 ± 0.324a	1.639 ± 0.225b

The different *letter* a and b behind the data means significant (*p* < 0.05) differences between the groups

### Overview of assembly

We obtained 15.8 GB of clean reads from the metagenomic sequencing. After cleavage into 47 bp-Kmers, we subsequently gained 122,977 and 40,775 contigs (> 500 bp) from the scaffolds of S_1_ and S_2_, respectively. Statistical information of contigs (Table 2) indicated that these assemblies were sufficient for our study [55]. We obtained 204,739 and 68,701 ORFs via MetaGene Annotator (http://metagene.cb.k.u-tokyo.ac.jp/) from the contigs of S_1_ and S_2_, respectively. All ORFs with a length over 100 bp were subquently translated into amino acids. S_1_ had more ORFs when compared to S_2_, which consisted with the distribution of contigs.

**Table 2:** Summary of the metagenomic sequencing

Category	S_1_	S_2_
Raw reads	100,895,940	94,080,764
Clean reads	96,712,790	90,359,739
Assembled contigs	122,977	40,775
Largest contig length (bp)	47,750	144,494
Contig_N50 length^a^ (bp)	1014	949
Contig_N90 length^b^ (bp)	553	548
Predicted ORFs	204,739	68,701

^a^Contig_N50 length, the length of the smallest contig in the set of largest contigs that have a combined length that represents at least 50% of the assembly [74]

^b^Contig_N90 length, the length of the smallest contig in the set of largest contigs that have a combined length that represents at least 90% of the assembly [74]

### COG annotation and analysis

**Fig. 1:**
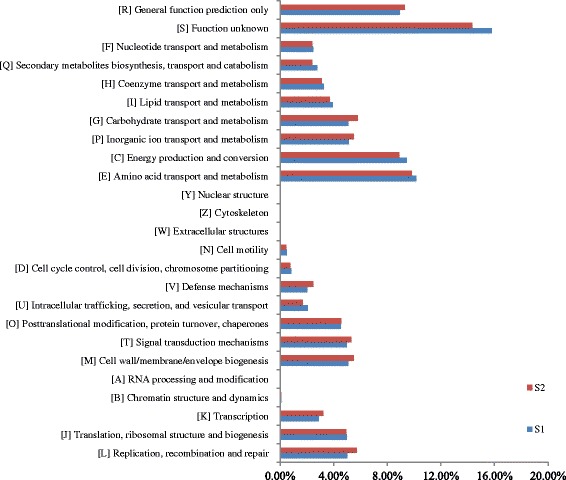
Distribution of predicted reads in the COG classification

The taxonomic assignments of the two samples were obtained by blast against NCBI-nt database. Notably, 39.8% of the predicted ORFs identified in the two samples can be assigned to putative functions. The classification of the functional potential genes was subsequently conducted via COG analysis. Amino acid transport and metabolism (E), energy production and conversion (C), general function prediction only (R) and function unknown (S) were the dominant functions among the 25 categories (Fig. 1). We summarized the results into four categories: information storage and processing (cluster I); cellular processes and signaling (cluster II); metabolism (cluster III); and poorly characterized function (cluster IV). Notably, cluster III was predominant in both samples and related to growth of the microbial community. However, the relative abundance of these metabolic genes in S_1_ was observably lower than in S_2_. Cluster IV was the next most abundant in both samples, followed by cluster II and I. In S2, the relative abundance of cluster III was higher than that of S_1_. The percentage of reads assigned to cell motility, cell cycle control, cell division, and chromosome partitioning were the lowest (≤ 1.0%)among the two samples. These sequencing data indicated that Cd-contamination inspired the metabolism of the microorganisms, as demonstrated by increase in relative abundance of metabolic genes detected in the Cd-polluted soil.

### Taxonomic composition of the microbial communities

**Fig. 2:**
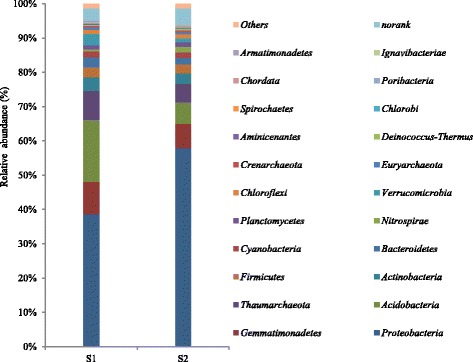
Relative abundances of all phyla in soil samples (*Others* < 0.10%)

The predicted ORFs with a length over 100 bp was selected and translated into amino acid sequence via the NCBI ORF finder, then annotated by BLASTP search (BLAST Version 2.2.28+) against NCBI-nr database [56] at the optimized E-value threshold of 1e-5. According to the corresponding taxonomic information in NCBI-nr database, we could obtain the taxonomy annotation and abundance of the species derived from the two samples. We assigned the classification confidence threshold in a lineage without classified information in the database as “*unclassified*”. We assigned “*norank*” to all sequences that could not be exactly classified into any known group at a taxonomic level. We classified the microorganisms with a relative abundance lower than 0.10% as “*Others*” (Fig. 2).

At phylum level, we summarized 77 taxa (apart from the *norank*) were from the two soil samples. According to the annotation, we defined *Proteobacteria*, *Gemmatimonadetes*, *Thaumarchaeota* and *Acidobacteria* as the dominant phyla in both S_1_ and S_2_, accounting for over 75% of the total population (Fig. 2). *Proteobacteria* was the most abundant phylum in both samples, comprising of 38.56% of all phyla in S_1_ and 57.85% in S_2_. These bacteria are important in soil, providing some basic functions related to the biogeochemical cycle. The *Proteobacteria* in the two samples embraced *Alphaproteobacteria*, *Betaproteobacteria*, *Gammaproteobacteria*, *Deltaproteobacteria* and *Epsilonproteobacteria*. Among them, *Betaproteobacteria* was the most abundant in the two soil samples. *Acidobacteria* was the second abundant phylum in S_1_ (18.13%), but was less abundant in S_2_ (6.30%). The high abundance of *Proteobacteria*, *Gemmatimonadetes*, *Thaumarchaeota* and *Acidobacteria* indicated that these phyla played key roles in the soil bacterial community.

**Fig. 3:**
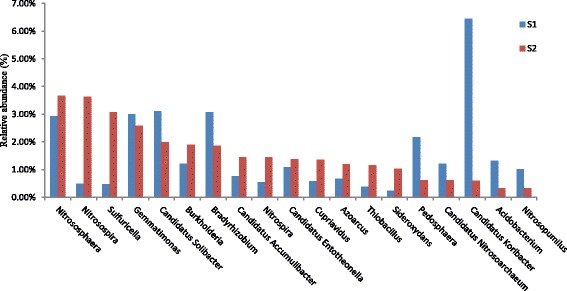
Relative abundances of dominant genera in soil samples

At the genus level, we obtained a total of 1736 genera (apart from the *norank*) from the two samples. Among of the whole genera, there were 1331 genera exist in both samples. There were 174 genera (apart from the *norank*) with a relative abundance greater than 0.10% of the total microorganisms in at least one of both samples. Figure 3 demonstrated the primary genera that each accounted for over 1.00% of the population in one of the two samples. The primary genera of S_1_ (*n* = 11) and S_2_ (14) make up close 30% of the whole soil community. *Candidatus Koribacter* (6.44%), *Candidatus Solibacter* (3.11%) and *Bradyrhizobium* (3.07%) were the three most abundant genera in S_1_. The taxonomic analysis showed that the bacteria belonged to *Nitrososphaera*, *Nitrosospira*, *Sulfuricella*, *Gemmatimonas*, *Candidatus Solibacter*, *Burkholderia*, *Bradyrhizobium*, *Candidatus Accumulibacter*, *Nitrospira*, *Candidatus Entotheonella*, *Cupriavidus*, *Azoarcus*, *Thiobacillus* and *Sideroxydans*, respectively. These bacteria accounted for 1.04% to 3.66% of the total community in S_2_ (Fig. 3), indicating that a majority of the core microbial community in soil responded to the Cd contamination. *Nitrososphaera* (3.66%), *Nitrosospira* (3.63%) and *Sulfuricella* (3.06%) were the three most abundant genera in S_2_. The total relative abundance of the three dominant genera in their own corresponding group accounted for over 10% of the entire 1736 detected genera. According to the distribution of the soil microbes at the phylum and genus level, we found that the abundance of microbial species in S1was higher than that in S2. Furthermore, the species diversity of S1 was greater than that of S2. The results showed that Cd-contamination decreased the microbial species diversity of the soil, but made the composition of microorganisms in the soil more concentrated.

### KEGG function annotation and analysis

Apart from phylogenic insights, metagenomic analysis also provided an opportunity to assess the functional potentials associated with the soil microbial community. We blasted the predicted genes against KEGG GENES via BLAST search (BLAST Version 2.2.28+, http://blast.ncbi.nlm.nih.gov/Blast.cgi) at the optimized threshold of 1e-5. Then, we conducted the functional annotation of genes via KOBAS 2.0 according to the blast results. Results indicated that the unpolluted soil (S_1_) has more genes, pathways, modules, orthologies and enzymes involved in the metabolic pathways of microbial communities than the Cd-contaminated soil (S_2_) (Table 3).

**Table 3:** Summary of the KEGG annotation results

Annotation categories	KEGG Genes	KEGG Pathway	KEGG Module	KEGG Orthology	Enzyme
S_1_	63,026	230	376	3680	1469
S_2_	21,622	213	331	2978	1248

**Fig. 4:**
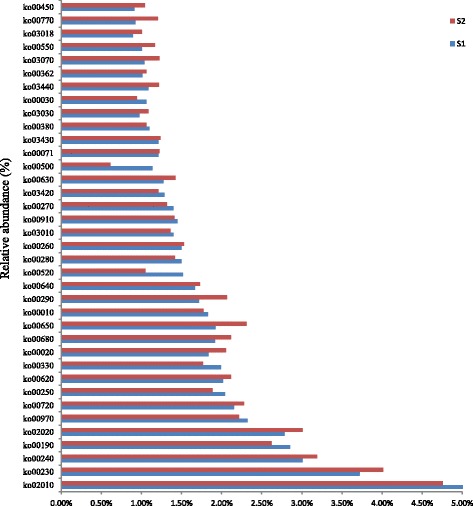
Relative abundance of dominant KEGG pathways (> 1.00%) in the two soil samples

**Fig. 5:**
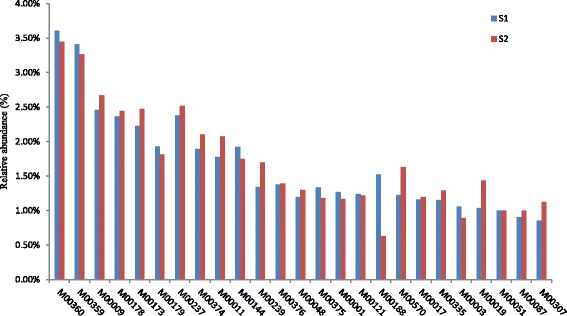
Relative abundance of dominant KEGG modules (> 1.0%) in the two soil samples

We have defined dominant pathway as those with a relative abundance greater than 1.00% of the total observed pathways. Then we obtained 37 dominant pathways in the two soil samples (Fig. 4). Among the 37 dominant pathways, the relative abundance of twenty-one KEGG pathways in the Cd-contaminated soil was higher than in the non-contaminated soil. There were 230 pathways determined in the two samples and the functions were depicted in the additional file. The most abundant pathway observed in the two samples was ko02010, which could be assigned to ABC transporters (Fig. 4 and Table 4). The relative abundance of ko02010 in S_1_ was higher than that in S_2_. But the relative abundances of some modules belonging to ko02010 were increased in S_2_ compared to S_1_, such as M00239 (Fig. 5). The ABC transporters could transport amino acids, lipids, lipid sugar, peptides and inorganic ions such as metal ions to the outside of the cells [56], and some of them also had a particular import function [57]. All the 37 dominant pathways were involved in some detoxification compound metabolisms (ko00230, ko00250), such as organic acid (ko00280), soluble amino acids (ko00330), fatty acid (ko00071), proteins and selenocompounds (ko00450), which could combine with Cd and decrease its biological toxicity [58, 59]. The collective abundance of the 37 dominant pathways accounted for almost 60% of the entire 230 pathways in S_1_ and 55% of the 213 pathways in the Cd-polluted soil (S_2_)_._ This indicated that Cd contamination can inhibit part of the KEGG pathways in the soil microorganisms. Meanwhile we also found that the relative abundances of other pathways (about 45% of the total pathways) in S_2_ increased when compared to S_1_, indicating that these pathways have the potential to help microorganisms cope with cadmium stress.

**Table 4:** Definition of the KEGG pathways (> 1.00%) in the two soil samples

Pathway	Definition
ko02010	ABC transporters
ko00230	Purine metabolism
ko00240	Pyrimidine metabolism
ko00190	Oxidative phosphorylation
ko02020	Two-component system
ko00970	Aminoacyl-tRNA biosynthesis
ko00720	Carbon fixation pathways in prokaryotes
ko00250	Alanine, aspartate and glutamate metabolism
ko00620	Pyruvate metabolism
ko00330	Arginine and proline metabolism
ko00020	Citrate cycle (TCA cycle)
ko00680	Methane metabolism
ko00650	Butanoate metabolism
ko00010	Glycolysis/Gluconeogenesis
ko00290	Valine, leucine and isoleucine biosynthesis
ko00640	Propanoate metabolism
ko00520	Amino sugar and nucleotide sugar metabolism
ko00280	Valine, leucine and isoleucine degradation
ko00260	Glycine, serine and threonine metabolism
ko03010	Ribosome
ko00910	Nitrogen metabolism
ko00270	Cysteine and methionine metabolism
ko03420	Nucleotide excision repair
ko00630	Glyoxylate and dicarboxylate metabolism
ko00500	Starch and sucrose metabolism
ko00071	Fatty acid metabolism
ko03430	Mismatch repair
ko00380	Tryptophan metabolism
ko03030	DNA replication
ko00030	Pentose phosphate pathway
ko03440	Homologous recombination
ko00362	Benzoate degradation
ko03070	Bacterial secretion system
ko00550	Peptidoglycan biosynthesis
ko03018	RNA degradation
ko00770	Pantothenate and CoA biosynthesis
ko00450	Selenocompound metabolism

In order to investigate the precise functions of the entire 230 pathways, we divided the entire pathways into 399 different functional modules. Almost 90% of the total pathways relative abundances in S_2_ were lower than those in S_1_, which indicated that Cd-contamination could affect the majority of the modules. There were 25 dominant modules (Fig. 5), as defined by the relative abundance greater than 1.00% of the whole modules in one or both of the samples. M00360 and M00359 were the two most abundant modules observed in the samples, and they were components of the ko00970 pathway, which was assigned to aminoacyl-tRNA bio-synthesis (Table 5). The relative abundance of the 25 dominant modules made up over 40% of the total modules identified in the two samples. M00009, M00011, M00017, M00019, M00048, M00051, M00087, M00173, M00178, M00237, M00239, M00307, M00335, M00374, M00376 and M00570 were the dominant modules in the Cd-contaminated soil. These sixteen (64%) of the dominant modules involved in the Cd-contaminated soil were of a higher relative abundance than those in the unpolluted soil, which indicated that these modules had a potential role in microbial resistance or tolerance to cadmium. Nine of the dominant modules involved in the unpolluted soil were of a higher relative abundance than those detected in Cd-contamination soil, suggesting that most of the dominant modules were inhibited under the Cd contamination. The results of the metabolism module analysis revealed that the relative abundance of some metabolisms decreased significantly in Cd-polluted soil.

**Table 5:** Definition of dominant KEGG modules (> 1.0%) in the two soil samples

Module	Definition
M00009	Citrate cycle (TCA cycle, Krebs cycle) [PATH:map01200 map00020]
M00178	Ribosome, bacteria [PATH:map03010]
M00173	Reductive citrate cycle (Arnon-Buchanan cycle) [PATH:map01200 map00720]
M00237	Branched-chain amino acid transport system [PATH:map02010] [BR:ko02000]
M00374	Dicarboxylate-hydroxybutyrate cycle [PATH:map01200 map00720]
M00011	Citrate cycle, second carbon oxidation, 2-oxoglutarate = > oxaloacetate [PATH:map01200 map00020]
M00239	Peptides/nickel transport system [PATH:map02010]
M00376	3-Hydroxypropionate bi-cycle [PATH:map01200 map00720]
M00048	Inosine monophosphate biosynthesis, PRPP + glutamine = > IMP [PATH:map00230]
M00570	Isoleucine biosynthesis, threonine = > 2-oxobutanoate = > isoleucine [PATH:map01230 map00290]
M00017	Methionine biosynthesis, aspartate = > homoserine = > methionine [PATH:map01230 map00270]
M00335	Sec (secretion) system [PATH:map03070]
M00019	Valine/isoleucine biosynthesis, pyruvate = > valine / 2-oxobutanoate = > isoleucine [PATH:map01210 map01230 map00290]
M00051	Uridine monophosphate biosynthesis, glutamine (+ PRPP) = > UMP [PATH:map00240]
M00087	beta-Oxidation [PATH:map01212 map00071]
M00307	Pyruvate oxidation, pyruvate = > acetyl-CoA [PATH:map01200 map00010 map00020 map00620]

**Fig. 6:**
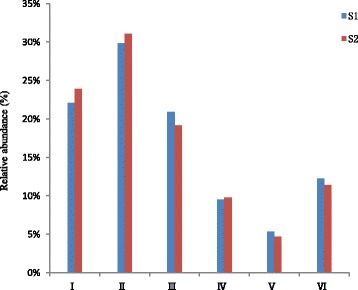
The relative abundance of enzymes associated with the two soil samples

In order to completely understand the metabolism variation in the two soil samples, we summarized the enzymes involved in all the identified 230 pathways, then, we achieved 1581 enzymes from the metagenomic sequencing. According to the International Enzymatic Commission classification system, the enzymes associated with these soil samples were classified into six categories: oxidoreductase (I), transferase (II), hydrolase (III), lyase (IV), isomerase (V) and ligase or synthetase (VI). The relative abundance of these enzymes was calculated via BLAST against the KEGG database (Fig. 6). Transferase was the most abundant enzymatic category, followed by oxidoreductase. Isomerase was the least abundant enzyme observed in the samples. The relative abundance of hydrolase, isomerase and ligase in S2 was lower than that of S1, but the relative abundance of oxidoreductase, transferase, and lyase in S_2_ was higher than that in S_1_, which indicated that these enzymes had a function potential of tolerance to Cd-contamination and could help the soil microorganisms endure Cd stress.

## Discussion

In this study, high-throughput metagenomic sequencing provided a powerful strategy to investigate the microbial community structure and functional potential associated in Cd-contaminated soil. Previous studies reported that *Nitrosospira*-like organisms were the major nitrifiers of the community that may possess cadmium tolerance [60, 61]. By characterizing the abundances of genes belonging to these genera, metagenomic sequencing revealed the variations of microbial abundance and diversity under different environmental conditions. For example, the relative abundances of genera belonging to *Nitrosospira* and *Sulfuricella* in S_1_ were 0.49% and 0.47%, respectively (Fig. 3), while their relative abundances in S_2_ increased to 3.63% and 3.06%, respectively. These results revealed that Cd contamination could increase the relative abundances of some microorganisms with a potential tolerance to heavy metal, and subsequently decreased the abundance of other microorganisms sensitive to heavy metals.

All of the core microorganisms were Gram-negative *Proteobacteria*, and most of them were related to the nitrogen cycle, which was an important process within the soil microbial community. It was reported that *Sulfuricella* and *Thiobacillus* mainly caused denitrification and dissimilatory sulfate reduction [62]. *Sideroxydans* belonged to a kind of iron oxide bacteria, *Candidatus Accumulibacter* was a phosphorus accumulating bacteria [63], and *Cupriavidus* was capable of a tolerance to copper [64]. All functions involved in these bacteria contributed to the tolerance of microorganisms to heavy metals [65–67].

**Fig. 7:**
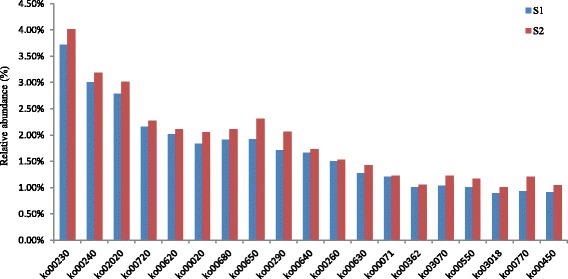
The relative abundances of pathways associated with the samples

We found that most of the COG annotations were involved in microbial metabolism (Fig. 1), and the relative abundance of cluster III (metabolism) was the highest among the four clusters but the relative abundance of metabolic genes in S_1_ was lower than that in S_2_. This indicated that Cd-contamination increased the activity of microorganisms in the soil. An increase in the relative abundance of metabolism genes suggested that the corresponding bacteria possessed a potential tolerance to Cd, and carried out normal metabolism in Cd-contaminated soil. The most abundant pathway among the entire 230 pathways affected by the Cd contamination was ko02010, which was assigned to ABC transporter. The current research on microbial ABC transporters showed that it involved in many biological functions, including bacterial drug resistance, pheromone secretion, mitochondria biological function and detoxification of heavy metals, etc. [68–70]. It was reported that ABC transporter played an important role in heavy metal detoxification, and was regarded to be resistant to Cd contamination in *Schizosaccharomyces pombe* and *Saccharomyces cerevisae* [68–70]. It was generally acknowledged that the mechanism of microbes coping with the heavy metal could be classified into two modes: in vitro and in vivo [71, 72]. Microbial organism could synthesis and secrete some chelates to forming co-precipitation with heavy metals under heavy metal stress. Then, these co-precipitations were prevented entering into the cells by microbial membrane. On the other hand, the organic acid or some proteins in vivo could combine with the heavy metals entered into the cells to form complex precipitation and detoxify the heavy metal poisoning through some enzymatic reaction or fixing them in the low active part [73]. Most of the dominant pathways whose abundance increased in the Cd contaminated soil were related to the metabolism, biosynthesis and degradation of amino acids, fatty acids and nucleotides, suggesting that these metabolic activities were related to Cd tolerance in the microorganisms or could help them survive from Cd-contamination at least (Fig. 7 and Additional file 1). Our results revealed that some soil microorganisms had a potential function in heavy metal tolerance and provided a new way to explore indigenous genera capable of bio-remediation.

The metagenomic analysis used in our experiment indicated that the method was adequate to assess the influence of cadmium pollution on microbial community and functional potential. The soil microorganisms were quite abundant, and many of them possessed numerous physiological functions. A stable microbial community is a critical factor to keep the bioavailability of soil. The total metagenomic sequences were assigned to 77 different phyla and 1736 genera. Among them, *Proteobacteria*, *Gemmatimonadetes*, *Thaumarchaeota* and *Acidobacteria* were the most common phyla in the samples, and comprised more than 75% of the total population in each sample. The results suggested that Cd-contamination had the potential to decrease the taxonomic species of microbes in soil, and further changed the soil microbial composition. It was also revealed that the functional pathways involved in the soil changed with microbial structure variation, many of which were related to the heavy metal tolerance of the soil microbes. These findings illustrated that Cd-contaminated soil was potential resource for exploring cadmium resistant or tolerant bacteria. In our study, all the core microorganisms were Gram-negative bacteria, belonging to *Proteobacteria*, with most of them being related to the nitrogen cycle and heavy metal tolerance. These bacteria and their physiological functions were important for the soil microbial community to cope with cadmium, as well as the stability and biological availability of the soil.

## Conclusion

Our research indicates that Cd-contamination has the potential to decrease the taxonomic species of microbes in soil, and further change the soil microbial composition. The functional pathways involved in the soil change with microbial structure variation, many of which are related to the heavy metal tolerance of the soil microbes. The Cd-contaminated soil is a potential resource for exploring cadmium resistant or tolerant bacteria.

## Additional file

Additional file 1: Definition of the KEGG pathways in the two soil samples. (DOCX 22 kb)

## Abbreviations

AAS: Atomic absorption spectroscopy
AM: *Arbuscular Mycorrhizal*
Cd: Cadmium
COG: Cluster of orthologous groups
ICP-MS: Inductively coupled plasma mass spectrometry
KEGG: Kyoto Encyclopedia of Genes and Genomes
ORFs: Open reading frames
PDB: Protein Data Bank
PIR: Protein Information Resource
PRF: Protein Research Foundation
SOM: Soil organic matter
TK: Total potassium
TN: Total nitrogen
TP: Total phosphorous
