# Supplementary material for: Metagenomic analysis of microbial community and function involved in cd-contaminated soil
Source: BMC Microbiol. 2018 Feb 13;18:11. doi: 10.1186/s12866-018-1152-5 (PMC5812035; doi:10.1186/s12866-018-1152-5)
Supplement: Supplementary file 1 — Definition of the KEGG pathways in the two soil samples. (DOCX 22 kb) [file 12866_2018_1152_MOESM1_ESM.docx]

**Metagenomic Analysis of Microbial Community and Function Involved in Cd-Contaminated Soil**

Gang Feng^a, 1^, Tian Xie^a, 1^, Xin Wang^a^, Jiuyuan Bai^a^, Qinglong Yan^a^, Tao Wei^a^, Lin Tang^b^, Hai Zhao^c^, Wei Wei^a^, Maolin Wang^a^, Yun Zhao^a,^ *

^a^ *Key Laboratory of Bio-Resource and Eco-Environment of Ministry of Education, College of life sciences, Sichuan University, Chengdu 610065, China*

^b^ *Tibet Academy of Agriculture and Animal Husbandry Sciences, Lhasa 850002, China*

^c^ *Chengdu Institute of Biology, Chinese Academy of Sciences, Chengdu 610041, China*

E-Mail: fenggangscu@foxmail.com (G. Feng); Xietianxietian12@qq.com (T. Xie); 2015222040102@stu.scu.edu.cn (X. Wang); 137304580@qq.com (J. Bai); yanqinglong@outlook.com (Q. Yan); wt11099@163.com (T. Wei); tanglinls@163.com (L. Tang); zhaohai@cib.ac.cn (H. Zhao); wwxfbxw@163.com (W. Wei); mlwang@scu.edu.cn (M. Wang).

^*^ Corresponding author. Address: College of Life Sciences, Sichuan University, No. 24 South Section 1, Yihuan Road, Chengdu 610065, China. Tel.: (+86-28) 85418776. Fax: (+86-28)85412485.

E-mail address: zhaoyun@scu.edu.cn (Y. Zhao).

^1^ Equal contributors.

**Additional file:** Definition of all the KEGG pathways (>1.00%) in the two soil samples

| Pathway | Definition |
| --- | --- |
| ko00040 | Pentose and glucuronate interconversions |
| ko00051 | Fructose and mannose metabolism |
| ko00053 | Ascorbate and aldarate metabolism |
| ko00062 | Fatty acid elongation in mitochondria |
| ko00072 | Synthesis and degradation of ketone bodies |
| ko00120 | Primary bile acid biosynthesis |
| ko00130 | Ubiquinone and other terpenoid-quinone biosynthesis |
| ko00140 | Steroid hormone biosynthesis |
| ko00250 | Alanine, aspartate and glutamate metabolism |
| ko00260 | Glycine, serine and threonine metabolism |
| ko00270 | Cysteine and methionine metabolism |
| ko00280 | Valine, leucine and isoleucine degradation |
| ko00290 | Valine, leucine and isoleucine biosynthesis |
| ko00311 | Penicillin and cephalosporin biosynthesis |
| ko00330 | Arginine and proline metabolism |
| ko00361 | Chlorocyclohexane and chlorobenzene degradation |
| ko00400 | Phenylalanine, tyrosine and tryptophan biosynthesis |
| ko00430 | Taurine and hypotaurine metabolism |
| ko00440 | Phosphonate and phosphinate metabolism |
| ko00450 | Selenocompound metabolism |
| ko00460 | Cyanoamino acid metabolism |
| ko00471 | D-Glutamine and D-glutamate metabolism |
| ko00472 | D-Arginine and D-ornithine metabolism |
| ko00513 | Various types of N-glycan biosynthesis |
| ko00520 | Amino sugar and nucleotide sugar metabolism |
| ko00522 | Biosynthesis of 12-, 14- and 16-membered macrolides |
| ko00523 | Polyketide sugar unit biosynthesis |
| ko00524 | Butirosin and neomycin biosynthesis |
| ko00531 | Glycosaminoglycan degradation |
| ko00540 | Lipopolysaccharide biosynthesis |
| ko00562 | Inositol phosphate metabolism |
| ko00564 | Glycerophospholipid metabolism |
| ko00500 | Starch and sucrose metabolism |
| ko00590 | Arachidonic acid metabolism |
| ko00592 | alpha-Linolenic acid metabolism |
| ko00603 | Glycosphingolipid biosynthesis-globo series |
| ko00604 | Glycosphingolipid biosynthesis-ganglio series |
| ko00624 | Polycyclic aromatic hydrocarbon degradation |
| ko00625 | Chloroalkane and chloroalkene degradation |
| ko00630 | Glyoxylate and dicarboxylate metabolism |
| ko00660 | C5-Branched dibasic acid metabolism |
| ko00670 | One carbon pool by folate |
| ko00710 | Carbon fixation in photosynthetic organisms |
| ko00720 | Carbon fixation pathways in prokaryotes |
| ko00760 | Nicotinate and nicotinamide metabolism |
| ko00770 | Pantothenate and CoA biosynthesis |
| ko00860 | Porphyrin and chlorophyll metabolism |
| ko00900 | Terpenoid backbone biosynthesis |
| ko00901 | Indole alkaloid biosynthesis |
| ko00903 | Limonene and pinene degradation |
| ko00940 | Phenylpropanoid biosynthesis |
| ko00944 | Flavone and flavonol biosynthesis |
| ko00945 | Stilbenoid, diarylheptanoid and gingerol biosynthesis |
| ko00950 | Isoquinoline alkaloid biosynthesis |
| ko00960 | Tropane, piperidine and pyridine alkaloid biosynthesis |
| ko00980 | Metabolism of xenobiotics by cytochrome P450 |
| ko00982 | Drug metabolism-cytochrome P450 |
| ko00983 | Drug metabolism-other enzymes |
| ko01040 | Biosynthesis of unsaturated fatty acids |
| ko01053 | Biosynthesis of siderophore group nonribosomal peptides |
| ko01055 | Biosynthesis of vancomycin group antibiotics |
| ko01056 | Biosynthesis of type II polyketide backbone |
| ko01057 | Biosynthesis of type II polyketide products |
| ko02060 | Phosphotransferase system (PTS) |
| ko03008 | Ribosome biogenesis in eukaryotes |
| ko03022 | Basal transcription factors |
| ko03450 | Non-homologous end-joining |
| ko04011 | MAPK signaling pathway - yeast |
| ko04020 | Calcium signaling pathway |
| ko04062 | Chemokine signaling pathway |
| ko04070 | Phosphatidylinositol signaling system |
| ko04120 | Ubiquitin mediated proteolysis |
| ko04141 | Protein processing in endoplasmic reticulum |
| ko04612 | Antigen processing and presentation |
| ko04614 | Renin-angiotensin system |
| ko04621 | NOD-like receptor signaling pathway |
| ko04622 | RIG-I-like receptor signaling pathway |
| ko04623 | Cytosolic DNA-sensing pathway |
| ko04626 | Plant-pathogen interaction |
| ko04666 | Fc gamma R-mediated phagocytosis |
| ko04712 | Circadian rhythm-plant |
| ko04810 | Regulation of actin cytoskeleton |
| ko04914 | Progesterone-mediated oocyte maturation |
| ko04920 | Adipocytokine signaling pathway |
| ko04964 | Proximal tubule bicarbonate reclamation |
| ko04973 | Carbohydrate digestion and absorption |
| ko04974 | Protein digestion and absorption |
| ko05014 | Amyotrophic lateral sclerosis (ALS) |
| ko05100 | Bacterial invasion of epithelial cells |
| ko05110 | Vibrio cholerae infection |
| ko05111 | Vibrio cholerae pathogenic cycle |
| ko05120 | Epithelial cell signaling in Helicobacter pylori infection |
| ko05130 | Pathogenic Escherichia coli infection |
| ko05142 | Chagas disease (American trypanosomiasis) |
| ko05150 | Staphylococcus aureus infection |
| ko05322 | Systemic lupus erythematosus |
| ko05410 | Hypertrophic cardiomyopathy (HCM) |
| ko00010 | Glycolysis / Gluconeogenesis |
| ko00020 | Citrate cycle (TCA cycle) |
| ko00030 | Pentose phosphate pathway |
| ko00052 | Galactose metabolism |
| ko00061 | Fatty acid biosynthesis |
| ko00071 | Fatty acid metabolism |
| ko00100 | Steroid biosynthesis |
| ko00190 | Oxidative phosphorylation |
| ko00195 | Photosynthesis |
| ko00230 | Purine metabolism |
| ko00232 | Caffeine metabolism |
| ko00240 | Pyrimidine metabolism |
| ko00253 | Tetracycline biosynthesis |
| ko00281 | Geraniol degradation |
| ko00300 | Lysine biosynthesis |
| ko00310 | Lysine degradation |
| ko00340 | Histidine metabolism |
| ko00350 | Tyrosine metabolism |
| ko00351 | DDT degradation |
| ko00360 | Phenylalanine metabolism |
| ko00312 | beta-Lactam resistance |
| ko00362 | Benzoate degradation |
| ko00363 | Bisphenol degradation |
| ko00364 | Fluorobenzoate degradation |
| ko00380 | Tryptophan metabolism |
| ko00401 | Novobiocin biosynthesis |
| ko00410 | beta-Alanine metabolism |
| ko00473 | D-Alanine metabolism |
| ko00480 | Glutathione metabolism |
| ko00510 | N-Glycan biosynthesis |
| ko00511 | Other glycan degradation |
| ko00521 | Streptomycin biosynthesis |
| ko00550 | Peptidoglycan biosynthesis |
| ko00561 | Glycerolipid metabolism |
| ko00565 | Ether lipid metabolism |
| ko00591 | Linoleic acid metabolism |
| ko00600 | Sphingolipid metabolism |
| ko00620 | Pyruvate metabolism |
| ko00621 | Dioxin degradation |
| ko00622 | Xylene degradation |
| ko00623 | Toluene degradation |
| ko00626 | Naphthalene degradation |
| ko00627 | Aminobenzoate degradation |
| ko00633 | Nitrotoluene degradation |
| ko00640 | Propanoate metabolism |
| ko00642 | Ethylbenzene degradation |
| ko00643 | Styrene degradation |
| ko00650 | Butanoate metabolism |
| ko00680 | Methane metabolism |
| ko00730 | Thiamine metabolism |
| ko00740 | Riboflavin metabolism |
| ko00750 | Vitamin B6 metabolism |
| ko00780 | Biotin metabolism |
| ko00785 | Lipoic acid metabolism |
| ko00790 | Folate biosynthesis |
| ko00791 | Atrazine degradation |
| ko00830 | Retinol metabolism |
| ko00906 | Carotenoid biosynthesis |
| ko00908 | Zeatin biosynthesis |
| ko00910 | Nitrogen metabolism |
| ko00920 | Sulfur metabolism |
| ko00930 | Caprolactam degradation |
| ko00941 | Flavonoid biosynthesis |
| ko00943 | Isoflavonoid biosynthesis |
| ko00965 | Betalain biosynthesis |
| ko00970 | Aminoacyl-tRNA biosynthesis |
| ko04210 | Apoptosis |
| ko04260 | Cardiac muscle contraction |
| ko04360 | Axon guidance |
| ko04510 | Focal adhesion |
| ko04512 | ECM-receptor interaction |
| ko04724 | Glutamatergic synapse |
| ko04725 | Cholinergic synapse |
| ko04742 | Taste transduction |
| ko04744 | Phototransduction |
| ko04916 | Melanogenesis |
| ko04976 | Bile secretion |
| ko04978 | Mineral absorption |
| ko05010 | Alzheimer's disease |
| ko05012 | Parkinson's disease |
| ko05016 | Huntington's disease |
| ko05020 | Prion diseases |
| ko04930 | Type II diabetes mellitus |
| ko04940 | Type I diabetes mellitus |
| ko04910 | Insulin signaling pathway |
| ko05133 | Pertussis |
| ko05143 | African trypanosomiasis |
| ko05144 | Malaria |
| ko05145 | Toxoplasmosis |
| ko05146 | Amoebiasis |
| ko05152 | Tuberculosis |
| ko05160 | Hepatitis C |
| ko05162 | Measles |
| ko05164 | Influenza A |
| ko05200 | Pathways in cancer |
| ko05210 | Colorectal cancer |
| ko05211 | Renal cell carcinoma |
| ko05215 | Prostate cancer |
| ko05219 | Bladder cancer |
| ko05222 | Small cell lung cancer |
| ko05416 | Viral myocarditis |
| ko05323 | Rheumatoid arthritis |
| ko05340 | Primary immunodeficiency |
| ko01051 | Biosynthesis of ansamycins |
| ko02010 | ABC transporters |
| ko02020 | Two-component system |
| ko02030 | Bacterial chemotaxis |
| ko02040 | Flagellar assembly |
| ko03010 | Ribosome |
| ko03013 | RNA transport |
| ko03015 | mRNA surveillance pathway |
| ko03018 | RNA degradation |
| ko03020 | RNA polymerase |
| ko03030 | DNA replication |
| ko03040 | Spliceosome |
| ko03050 | Proteasome |
| ko03060 | Protein export |
| ko03070 | Bacterial secretion system |
| ko03320 | PPAR signaling pathway |
| ko03410 | Base excision repair |
| ko03420 | Nucleotide excision repair |
| ko03430 | Mismatch repair |
| ko03440 | Homologous recombination |
| ko04110 | Cell cycle |
| ko04111 | Cell cycle-yeast |
| ko04112 | Cell cycle-Caulobacter |
| ko04113 | Meiosis-yeast |
| ko04115 | p53 signaling pathway |
| ko04122 | Sulfur relay system |
| ko04142 | Lysosome |
| ko04145 | Phagosome |
| ko04146 | Peroxisome |
| ko04150 | mTOR signaling pathway |
